# Effects of Shade and Planting Methods on the Growth of *Heracleum moellendorffii* and *Adenophora divaricata* in Different Soil Moisture and Nutrient Conditions

**DOI:** 10.3390/plants10102203

**Published:** 2021-10-17

**Authors:** Woo Bin Youn, Jonathan Ogayon Hernandez, Byung Bae Park

**Affiliations:** 1 Department of Environment and Forest Resources, Chungnam National University, Daejeon 34134, Korea; dbsdnqls95@cnu.ac.kr (W.B.Y.); johernandez2@up.edu.ph (J.O.H.); 2 Department of Forest Biological Sciences, University of the Philippines, Los Baños 4031, Philippines

**Keywords:** fertilization, interspecific competition, planting methods, shade, plant growth, soil moisture, understory

## Abstract

In this study, the interacting effects of shade and planting methods on the growth and competitive ability of two understory plants *Heracleum moellendorffii* Hance and *Adenophora divaricata* Franch. & Sav. were investigated under different soil moisture and nutrient conditions. One-year-old seedlings were subjected to different light levels (0%, 35%, and 55% shade) and planting methods (monoculture and mixed) under contrasting soil moisture (1.2 L/m^2^ and 2.3 L/m^2^ of water) and soil nutrient conditions (unfertilized and fertilized). Here, shading significantly improved the height growth of *H. moellendorffii* (10–20 cm increase) in unfertilized and fertilized plots and at high soil moisture conditions. Contrarily, *A. divaricata* seedlings planted singly at full sunlight yielded a higher aboveground biomass growth (8–17 g plant^−1^), compared with those shaded and intercropped seedlings (0.9–3.9 g plant^−1^). The increased competitiveness of *H. moellendorffii* suppressed the growth of *A. divaricata* across different light conditions when planted together. The amount of light, soil moisture, and nutrients and their interactions significantly affected the growth of the seedlings, resulting in asymmetric interspecific competition between the two species. Results provide us with a better understanding of the environmental factors affecting plant growth for forest farming in the understory.

## 1. Introduction

The growing demands for food to meet the need of the growing global populations has resulted in environmental pressures on forest lands, including forest clearing for commercial agriculture [1] and unsustainable exploitation of forest resources [2]. If these pressures continue, deforestation and negative impacts on forest ecosystems and services will likely become more evident in the future. Thus, studies on potential alternatives to increase agricultural production without exploiting forest lands and resources can significantly reduce the pressures or threats to our forest ecosystems.

One of the alternative approaches to reduce pressures on forest ecosystems is forest farming, which is defined as the cultivation of crops in the forest understory of either established or developing forests [3]. Its goal is to increase crop yields by cultivating plants that can grow under a forest canopy [4] and thereby maximize sustainable harvests of forests products [5]. In a few forests in the USA, forest landowners recognized that improving the diversity of plants for food and/or medicines in the forests can conserve biodiversity, provide ecosystem services, and improve forest management while providing small-scale forest landowners or communities with a good source of income [5,6]. A study also reported that local communities living near the forests in Nigeria rely on non-timber forest products, including herbs for household and commercial use [7]. To date, however, there is little published information on factors affecting crop yield and abundance in the forest understory. Considering the unique environment in the forest understory, compared with the typical agricultural lands, it is, therefore, necessary to enhance our understanding of the environmental factors affecting plant growth. Doing so will make forest farming more ecologically and socio-economically feasible.

Plant growth and abundance are predominantly influenced by the availability of light, soil nutrients, soil moisture, and substrates [8]. An experiment showed that plants grown in full sunlight produced more biomass, foliage, and allocated a larger proportion of their total production to belowground biomass than plants grown in 60–90% shade [9]. The authors also noted that shade-treated plants flowered 2–6 weeks later than those planted under full sunlight. A contrasting result was observed in the case of common bell pepper, i.e., a 30% shade level resulted in a significantly higher yield, compared with the open field condition [10]. Similarly, a study also reported that plants planted at 50% shade level resulted in a remarkably higher plant height, compared with control [11]. In an actual understory field experiment, plant growth responses to different light conditions varied considerably by seasonal variations in light availability and soil moisture gradient [12]. This is because, during the dry season, shady areas in the forest understory provide higher soil moisture availability and lower transpiration rate, compared with those in open canopies [13,14]. Plant growth and development may also be more rapid at more moist sites on low slope positions than those at high slopes [15]. In addition to light and soil moisture, plant growth is also greatly influenced by nutrient availability and fertilizer application [16,17]. For example, Casey et al. [18] concluded that the optimal conditions for “ngo gai” cultivation are at a combination of a nitrogen fertilization rate of >90 kg ha^−1^ and 40% shade. Moreover, understory plant growth is affected by competition for the limited availability of resources, particularly light, water, and nutrients [19]. A greenhouse study showed that when two understory species were planted together, the biomass growth varied considerably by soil treatment (i.e., higher biomass proportion was observed in nutrient-rich than nutrient-poor soil), whereas no significant difference was detected when the species was planted singly [20].

Research on the propagation techniques and growth environment of wild herbs has increased steadily in response to consumers’ demand and consumption. Two perennial understory herb species that merit further study are *Heracleum moellendorffii* Hance and *Adenophora divaricata* Franch. & Sav. In this study, they were selected based on their geographic distribution, habitat characteristics, and emerging economic potential as food and medicinal herbs. These species can survive at a wide range of environmental conditions in Korea, China, Mongolia, Japan, and Russia, including open forests, forest margins, streams, grasslands, and shaded valleys [21,22]. As per our observation, these species are commonly found growing together in forest understories in Korea, particularly in broadleaf and coniferous forests dominated by *Quercus* and *Pinus* species. Plants of *H. moellendorffii* and *A. divaricata* are harvested in the wild for food and medicines in the forest farming system in Gyeongsangbuk-do Province [23] and Jeollabuk-do Province in Korea [24]. For example, roots of *H. moellendorffii* are harvested by the locals to treat inflammatory human diseases [25]. *H. moellendorffii* grows in association with *Crypsinus hastatus* (Thunb.) Copel., *Penthorum chinense* Pursh and *Trachomitum lancifolium* (Russanouv) Pobed.in Daebudo and adjacent regions in Korea, characterized by having an average temperature of 12.5 °C and precipitation of 1275.9 mm [26]. *Adenophora divaricata* also grows in association with other arboreal and herbaceous species, such as *Larix olgensis* and *Adenophora lamarkii* in montane or subalpine slopes [27].

In Korea, wild vegetables are recognized as healthy vegetables due to their excellent nutritional value; hence, they are emerging as a promising source of income for mountain villagers. However, studies on a suitable growth environment for improving productivity and yield remain insufficient in many forest understory species. Thus, the effects of shade and planting methods on the growth and competitive ability of *H. moellendorffii* and *A. divaricata* were investigated under different soil moisture and nutrient conditions. We hypothesized that seedlings of both species will grow better in full sunlight with high soil moisture and nutrients because of the increased resource availability. We also hypothesized that competition will occur between the two species when planted together in low resource availability.

## 2. Results

### 2.1. Effects of Shade and Planting Methods on the Growth of Unfertilized and Fertilized H. moellendorffii and A. divaricata

**Figure 1:**
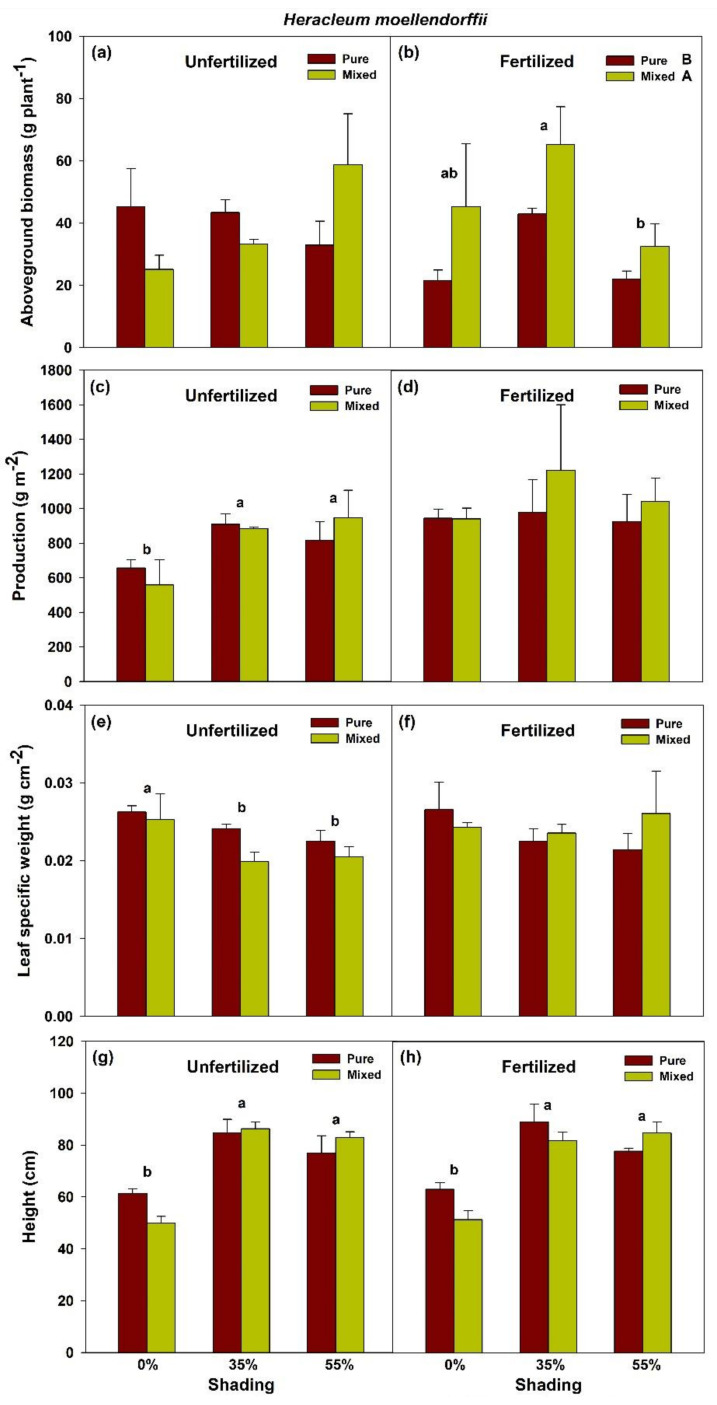
Aboveground biomass (**a**,**b**), shoot production (**c**,**d**), leaf-specific weight (**e**,**f**), and height growth (**g**,**h**) of pure *Heracleum moellendorffii* and mixed *Heracleum moellendorffii + Adenophora divaricata* in different light and fertilization treatments. Different lowercase and uppercase letters indicate statistical significance across shade treatments and between planting methods, respectively. Vertical bars show standard errors (*n* = 3).

In fertilized and unfertilized plots, there was no significant planting × shade interaction effect on the aboveground biomass, shoot production, leaf specific weight, and height of *H. moellendorffii* (Figure 1; Table S1). However, the main effect of shade on aboveground production in unfertilized plots was significantly different across treatments, i.e., higher in both 35% and 55% shade levels than full sunlight for both pure and mixed planting. Contrarily, all shade-treated seedlings in unfertilized plots had a significantly lower leaf-specific weight than those grown in full sunlight. The main effect of shade on height was also significantly different across treatments, i.e., 35 and 55% shade are equally higher than 0% shade (full sunlight) in both fertilized and unfertilized seedlings (Figure 1; Table S1). Moreover, the difference between pure and mixed planting treatments was also significant, such that mixed planting resulted in a significantly higher aboveground biomass, compared with the pure planting method in all shade treatments in fertilized plots.

**Figure 2:**
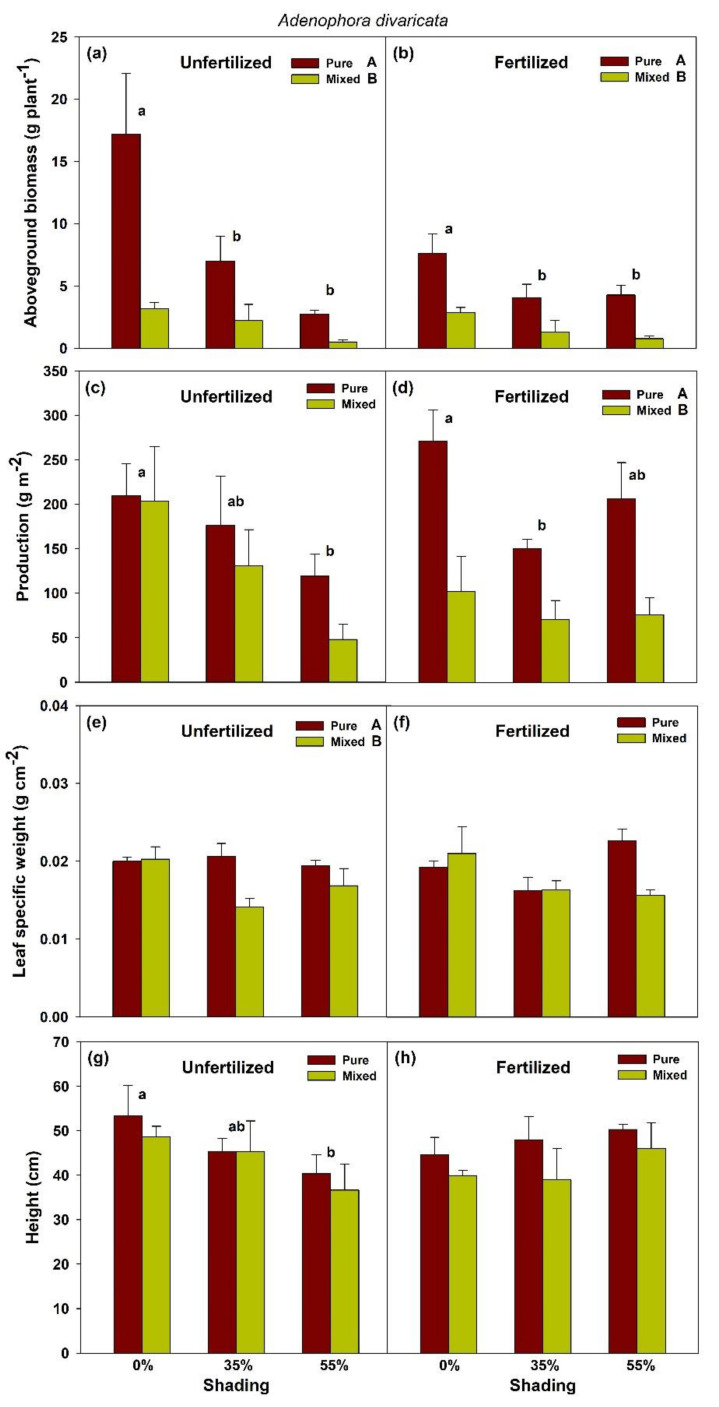
Aboveground biomass (**a**,**b**), shoot production (**c**,**d**), leaf-specific weight (**e**,**f**), and height growth (**g**,**h**) of pure *Adenophora divaricata* and mixed *Heracleum moellendorffii + Adenophora divaricata* in different light and fertilization treatments. Different lowercase and uppercase letters indicate statistical significance across shade treatments and between planting methods, respectively. Vertical bars show standard errors (*n* = 3).

For *A. divaricata*, the effects of planting × shade interaction were also generally not significant for all parameters measured, except for the aboveground biomass of unfertilized seedlings (Figure 2; Table S1). Seedlings planted singly at full sunlight had the highest aboveground biomass growth across shade and planting treatment combinations. We detected highly significant main effects of either shade or planting, such that seedlings of pure planting resulted in higher biomass for both unfertilized and fertilized seedlings, higher leaf-specific weight for only unfertilized, and higher production for only fertilized ones, compared with mixed planting. In terms of the main effect of shade, full sunlight yielded higher aboveground biomass and production, compared with those grown under shade conditions, for all unfertilized and fertilized seedlings. Full sunlight also gave the unfertilized seedlings higher height growth, compared with those shade-treated ones.

**Figure 3:**
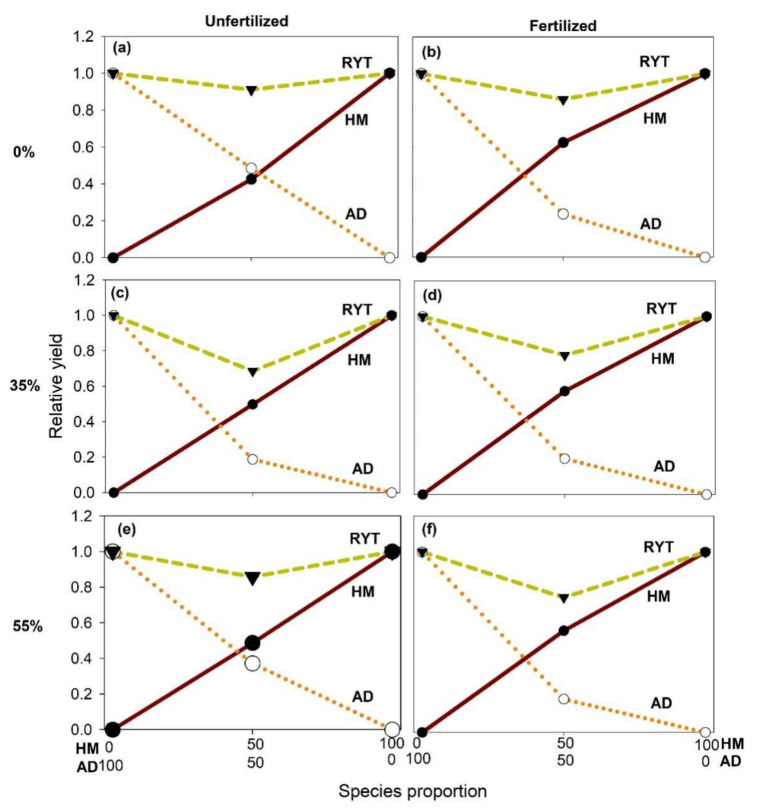
Relative yield total (RYT) of unfertilized and fertilized *Heracleum moellendorffii*. (HM) and *Adenophora divaricata* (AD) in 0% (**a**,**b**), 35% (**c**,**d**), and 55% (**e**,**f**) shade treatments.

Results also revealed that the relative yield total (RYT) of *H. moellendorffii* was higher at fertilized plots than at unfertilized plots regardless of shade treatments by 20–33% (Figure 3). In contrast, the RYT of *A. divaricata* decreased as light availability decreased, particularly at fertilized plots. Further, the RYT observed at all mixed planting plots was lower than one, suggesting that the yield per area of the two species may have been decreased due to interspecific competition.

### 2.2. Effects of Shade and Planting Methods on the Growth of H. moellendorffii and A. divaricata under High and Low Soil Moisture Conditions

**Figure 4:**
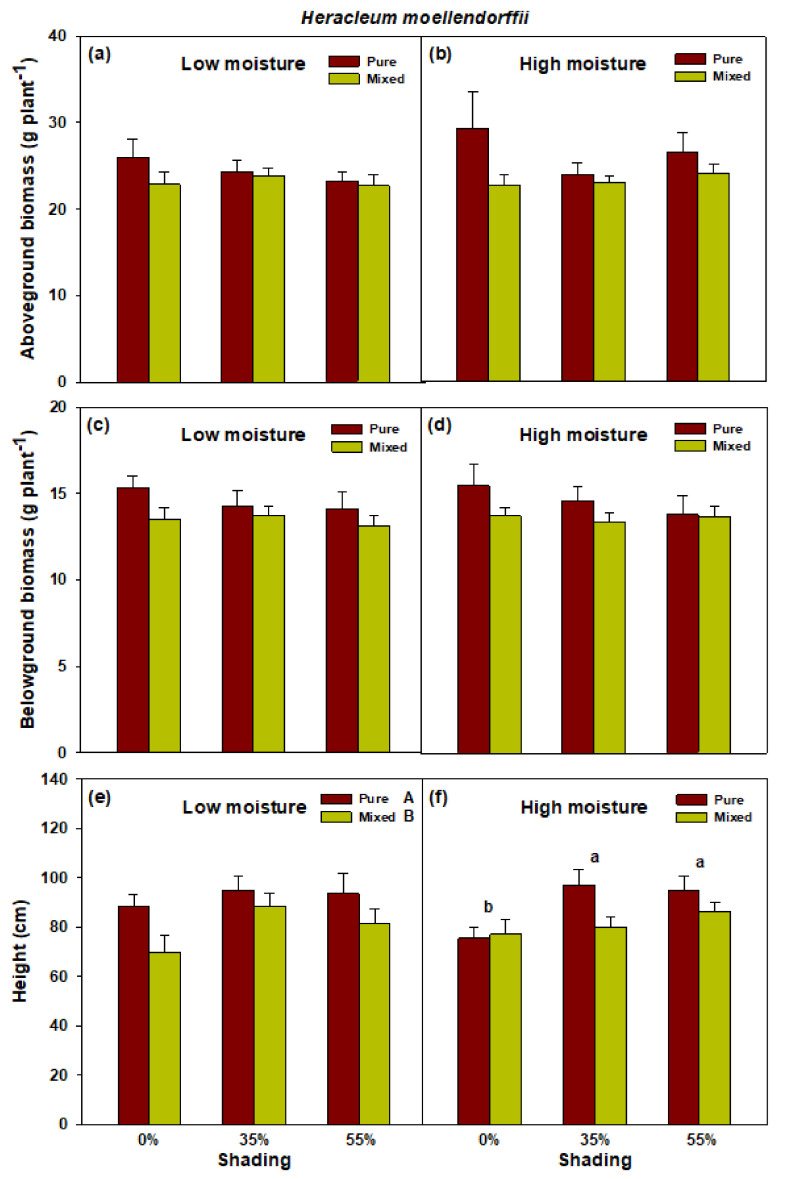
Aboveground biomass (**a**,**b**), belowground biomass (**c**,**d**), and height (**e**,**f**) of pure *Heracleum moellendorffii* and mixed *Heracleum moellendorffii + Adenophora divaricata* in different light and soil moisture conditions. Different lowercase and uppercase letters indicate statistical significance across shade treatments and between planting methods, respectively. Vertical bars show standard errors (*n* = 3).

There was no significant shade × planting interaction effect detected in *H. moellendorffii* for seedlings subjected to both low and high soil moisture conditions (Figure 4; Table S2). However, a significant main effect of planting method and shade on height growth of *H. moellendorffii* grown in low- and high-moisture conditions, respectively, was detected. Under high soil moisture conditions, height growth was generally higher in shade conditions (35–55%) than in full sunlight (0%), whereas no variation was detected across treatments for seedlings exposed to low soil moisture. The monoculture planting method under low soil moisture conditions resulted in significantly higher height growth, compared with that of the mixed method.

**Figure 5:**
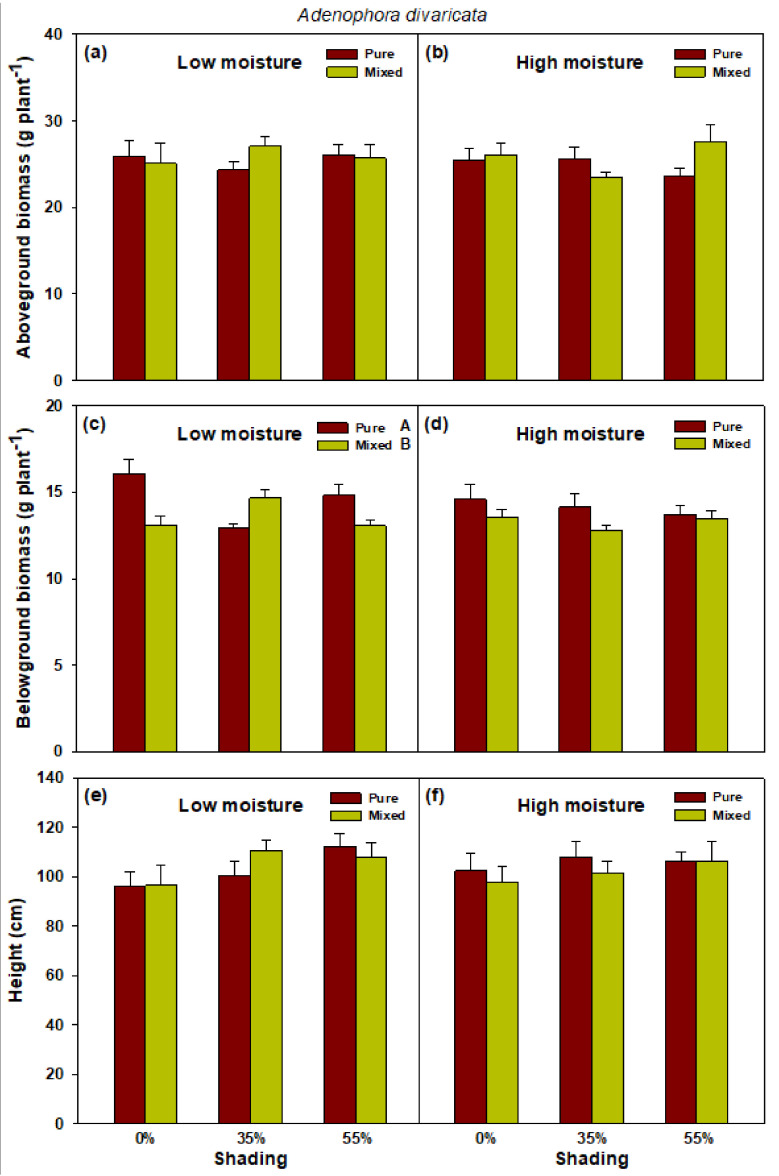
Aboveground biomass (**a**,**b**), belowground biomass (**c**,**d**), and height (**e**,**f**) of pure *Adenophora divaricata* and mixed *Heracleum moellendorffii + Adenophora divaricata* in different light and soil moisture conditions. Different lowercase and uppercase letters indicate statistical significance across shade treatments and between planting methods, respectively. Vertical bars show standard errors (*n* = 3).

A significant effect of interaction between shade and planting method on belowground biomass of *A. divaricata*, particularly those grown in a low soil moisture condition, was detected (Figure 5; Table S2). Belowground biomass growth was generally higher in the monoculture plot at full sunlight and 55% shade condition, compared with that in 35% shade.

## 3. Discussion

The growth and survival of plants are normally associated with their ability to intercept light efficiently [28] and the amount of nutrients and moisture available in soil [29,30]. One significant finding of this study was that shading improved the height growth of *H. moellendorffii* in both unfertilized and fertilized plots and aboveground production in unfertilized ones. Similarly, the seedling height of *H. moellendorffii* also responded positively to decreasing light availability at high-moisture conditions, and this agrees with the characteristics typical of shade-tolerant species. These results did not fully support our first hypothesis that seedlings will grow better in full sunlight with high soil moisture and nutrients because of the increased resource availability. However, our findings are consistent with the results reported in Kupers et al. [31], i.e., species that performed or grew well in shade suffered worse during severe dry months and vice versa. Generally, tolerances to low light and water availability are inversely correlated, such that plants that can grow at shade conditions may not survive under dry conditions because of the negative trade-off created between shade and drought tolerances [31,32,33]. No evidence in the literature about shade tolerance of the species has been reported to date, but a study has shown that an herb understory species from the same genus, *Heracleum sphondylium* L., had a moderate shade tolerance [33]. *H. moellendorffii* may have invested more assimilates for height growth/stem elongation to efficiently intercept and capture sunlight for photosynthesis amid low light availability regardless of nutrient conditions, and this may have resulted in higher aboveground production. Nagashima and Hikosaka [34] observed similar results, i.e., understory/lowered plants increased the rate of stem elongation of annual plant *Chenopodium album L*. as light availability decreased. It has long been regarded that such a response to shade or light availability is due to shade avoidance [35], which is common in many herbaceous plants [36]. Moreover, in this study, shade conditions resulted in a lower leaf specific weight, compared with that in full sunlight, particularly at unfertilized plots. This decrease in leaf-specific weight can be attributed to the increased leaf surface area because, generally, leaves must be as wide, flat, and thin as possible to absorb sufficient light [37].

The genus *Adenophora* is also generally known to thrive in moderate drought conditions and warm sunny positions in grassy and rocky slopes [38]. Here, seedlings from both species planted together at shade conditions yielded lower aboveground biomass, compared with those seedlings planted singly at full sunlight, especially in unfertilized plots. Based on our second hypothesis, the decrease in aboveground biomass of intercropped seedlings under shade conditions can be attributed to the interspecific competition that may have occurred between the two species when planted together in low resource availability (light and nutrients). Findings from our study are partially consistent with the study of Haan et al. [39], who reported that the yield of onion was highest in intraspecific competition and lowest in interspecific one with lettuce. The inability of *A. divaricata* to persist under shade and intercropped conditions may be attributed to the simultaneous interaction of the influence of shade and planting methods on resource allocation and interspecific resource competition. This can be supported by the decrease in RYT of the species when planted in combination with *H. moellendorffii* across light and nutrient conditions. The increased competitiveness of *H. moellendorffii* may have led to suppression of the growth of *A. divaricata*, suggesting that the former species may be a strong competitor of *A. divaricata* regardless of resource availability. The competition observed between *H. moellendorffii* and *A. divaricata* may be considered as asymmetric interspecific facilitation, where the mixed planting increases the yield of one species but causes a decrease in the other species. Several studies have already reported that competitive interactions among different species can have either a positive and negative effect on growth [40,41], and this is exemplified in our results.

Overall, results further imply that *H. moellendorffii* should dominate on nutrient-rich soils because of the ability to efficiently use whatever nutrients available to them through rapid height growth even at unfertilized soil, which may have given the species the advantage to compete over the other species. Additionally, the biomass growth of *A. divaricata* can be enhanced at full sunlight using a pure/monoculture planting method even at unfertilized soil. However, results may also imply that if *A. divaricata* is planted either singly or mixed at low soil moisture regardless of light availability, it may potentially proliferate and become invasive, which is typical of the genus *Adenophora*. This can be seen in the increased belowground biomass of *A. divaricata* seedlings grown even at low soil moisture, with either full sunlight or 55% shade condition when planted singly. Notably, when *A. divaricata* seedlings were planted together with *H. moellendorffii*, the belowground biomass of *A. divaricata* seedlings under low moisture was significantly higher in 35% shade than in the other shade conditions. Results imply that the interacting effects of soil moisture, shade, and planting methods may stimulate the root growth of the species, and may contribute to its invasive ability.

Considering that the two studied species are useful as food and medicinal herbs in some countries, including Korea, China, and Japan, the findings of this study can support sustainable forest management through forest farming in the understory [3,6]. Here, results revealed that *H. moellendorffii* can be planted at low light, with high levels of soil moisture and nutrients, and intercropped conditions, whereas *A. divaricata* has shown to be more suitable in full sunlight, low soil moisture and nutrients, and monoculture conditions. Planting of *H. moellendorffii* is, therefore, more suited in the forest understory in riparian areas, particularly in mountainous regions where most of the nutrients are usually leached and eroded towards riverbanks by water and wind on steep slopes [42]. In Korea, for instance, approximately 80% of the forested areas are found on steep terrain characterized by slopes greater than 40% [43]. Contrarily, *A. divaricata* may be suited in the understory in forest margins, where light and soil moisture and nutrients are relatively limited, compared with those in the middle and/or riparian areas in the forest.

## 4. Materials and Methods

### 4.1. Study Site and Experimental Materials

The study was composed of two experiments, which were conducted in two consecutive years (i.e., March–June 2017 and 2018) in a small vinyl-made phytotron (4 m × 2.0 m × 2.0 m) at Chungnam National University, Republic of Korea (36°22′N, 127°21′E). In 2017, we experimented on the interacting effects of different shade and planting methods on the growth of the select species in two different moisture conditions. In the same months in 2018, an experiment on the interacting effects of shade and planting methods in two soil nutrient conditions was conducted. During this period, the mean temperature was 13.45 °C, and the mean annual precipitation was 1334.8 mm. Nursery soil with 6.20 pH, 1.63% organic matter, 0.09% total N, and 102.30 mg kg^−1^ available P was used in this study (Table 1).

For this study, one-year-old seedlings of *Heracleum moellendorffii* Hance and *Adenophora divaricata* Franch. & Sav. were used as the test species. *H. moellendorffii* can grow up to one meter tall, in both broad-leaved mixed and coniferous forests, and is harvested from the wild for local use as food and medicine [21]. The species was already observed to grow in a wide range of environments, including open forests, forest margins, streams, grasslands, and shaded valleys [22,44]. There is little information about *A. divaricata* but its synonym *Adenophora divaricata* var. *manshurica* and other species in the genus were found to grow well in high altitude and high soil pH, low P_2_ O_5_, and sunny areas at the foot of mountains with abundant soil moisture as wild vegetables [45,46].

### 4.2. Experimental Design

In this experiment, a completely randomized design was employed. A total of 18 plots (4 m × 2.0 m), with a one-meter distance between plots, were established in the study site and subdivided into three subplots for each experiment. Two subplots were allotted to monoculture planting for each species and one subplot for the mixed planting of the two species (*A. divaricata* + *H. moellendorffii*). There were a total of 32 seedlings of each species for every monoculture plot and 65 seedlings for the mixed plot. The seedlings were planted in five and ten rows for monoculture and mixed plots, respectively, following a 20 cm distance between seedlings. Before treatment imposition, seedlings were first acclimatized for three weeks, in which all seedlings were watered daily.

For the 2017 experiment (Year 1), we used three levels of shade (i.e., 0%, 35%, and 55%) and two levels of moisture (i.e., low or high of water). The water was supplied twice per week through a drip irrigation system, with 1.2 L/m^2^ and 2.3 L/m^2^ for low- and high-moisture conditions, respectively. For the 2018 experiment (Year 2), we used two levels of fertilization (i.e., unfertilized and fertilized) and the same levels of shade treatments with Year 1. Only 2.3 L/m^2^ of water was applied to the unfertilized plots (control), whereas 2.3 L/m^2^ of water + 2 g L^−1^ of N-P-K (20:20:20) fertilizer (Scotts, Marysville, OH, USA), which was applied once a week via the drip irrigation system. During the course of the experiments, watering was performed twice a week.

### 4.3. Data Collection

At the end of the experiment, samples from each treatment were randomly selected from the center of the plot to measure the height, leaf-specific weight, above- and belowground biomass, and total aboveground production per unit area. The height was measured from the soil surface to the base of the leaves of the longest stem. Due to the relatively larger leaf size of *H. moellendorffii*, the leaves were cut into similar sizes (average: 17.26 cm^2^), and the weight was measured and converted into a unit weight. In the case of *A. divaricata*, one of the leaves in the third node from the bottom was selected, and the leaf area and weight were measured and converted into unit weights. The area of the leaves of *A*. *divaricata* was measured using an LI-3100 leaf area meter (LI-COR, Lincoln, NE, USA). Aboveground biomass was separated into leaves and stems for each treatment, dried at 65 °C, and weighed. The production per unit area was calculated by harvesting and weighing all samples from each treatment, and then the values obtained were divided by the area of the plot.

The relative yield total (RYT) was calculated to determine the effects of the change in light and nutrient availability on the interspecific competition and intraspecific competition of *A. divaricata* and *H. moellendorffii* [47,48]. The relative yield (RY) of each species was calculated using the following equation:

(1) $$\begin{array}{c} \mathrm{Relative}\;\mathrm{yield}\;\left(\mathrm{RY}\right)\;\mathrm{each}\;\mathrm{species}\;\mathrm{in}\;\mathrm{the}\;\mathrm{mixture}\\ =\frac{\mathrm{Yield}\;\mathrm{in}\;\mathrm{mixture}}{\mathrm{Yield}\;\mathrm{in}\;\mathrm{pure}\;\mathrm{stand}} \end{array}$$

(2) $$\begin{array}{l} \mathrm{Relative}\;\mathrm{yield}\;\mathrm{total}\;\left(\mathrm{RYT}\right)\\ =\mathrm{Relative}\;\mathrm{yield}\;\mathrm{of}\;H.\;moellendorffii\;\mathrm{in}\;\mathrm{the}\;\mathrm{mixture}\\ +\mathrm{Relative}\;\mathrm{yield}\;\mathrm{of}\;A.\;divaricata\;\mathrm{in}\;\mathrm{the}\;\mathrm{mixture} \end{array}$$

### 4.4. Statistical Analysis

For each species, we carried out a two-way analysis of variance (ANOVA) to analyze the effects of the main and interacting effects of the different shade and planting methods on the growth of the species for each soil moisture and fertilization condition. Duncan’s multiple range test was also run to evaluate comparisons among the treatments. All the statistical analyses were employed in R Statistical Package Software (version R-3.5.1, Boston, MA 02110-1301, USA), at α = 0.05 confidence level.

## 5. Conclusions

In this study, shade significantly increased the height growth of *H. moellendorffii* regardless of planting methods, particularly those grown in high soil moisture and nutrients. Contrarily, the aboveground biomass of *A. divaricata* was significantly suppressed by shading, particularly when planted with the other species without fertilizer. When planted together, the interspecific competitiveness of *H. moellendorffii* tended to be stronger than that of *A. divaricata* across light conditions. The amount of light, soil moisture, and nutrients and their interactions were shown to significantly affect the growth of the seedlings, resulting in asymmetric interspecific competition between species. The findings of this study provide us with a better understanding of the environmental factors affecting plant growth that are necessary to make forest farming in the understory more ecologically and socio-economically feasible and desirable.

## Figures and Tables

**Table 1:** Characteristics of nursery soil used in the study.

pH	Organic Matter (%)	Total N (%)	Available P (mg kg^−1^)	CEC (cmolc kg^−1^)	Exchangeable Cations (cmolc kg^−1^)			
					K^+^	Ca^2+^	Mg^2+^	Na^+^
6.2 (0.1)	1.63 (0.24)	0.09 (0.01)	102.3 (11.7)	4.35 (0.35)	0.18 (0.01)	0.06 (0.00)	3.51 (0.25)	0.80 (0.02)

## Data Availability

Data is contained within the article and a Supplementary Table S1 and Table S2.

## Supplementary Materials

The following are available online at https://www.mdpi.com/article/10.3390/plants10102203/s1, Table S1: *P*-values of two-way ANOVA for treatment effects and interaction on growth characteristics of unfertilized and fertilized *Heracleum moellendorffii* and *Adenophora divaricata*, Table S2: *P*-values of two-way ANOVA for treatment effects and interaction on growth characteristics of *Heracleum moellendorffii* and *Adenophora divaricata* grown in low and high soil moisture.
