# Supplementary material for: Effects of Shade and Planting Methods on the Growth of Heracleum moellendorffii and Adenophora divaricata in Different Soil Moisture and Nutrient Conditions
Source: Plants (Basel). 2021 Oct 17;10(10):2203. doi: 10.3390/plants10102203 (PMC8537555; doi:10.3390/plants10102203)
Supplement: Supplementary file 1 [file plants-10-02203-s001.zip › plants-1413813-supplementary.pdf]

Supplementary Materials

Table S1. P-values of two-way ANOVA for treatment effects and interaction on growth characteristics of unfertilized and fertilized *Heracleum moellendorffii* and *Adenophora divaricata*. Significant p-values are written in bold font.

		Heracleum moellendorffii		*Adenophora divaricata*	
Parameter	Factor	Unfertilized	Fertilized	Unfertilized	Fertilized
Aboveground biomass	Planting	0.854	**0.044**	**0.002**	**<0.001**
	Shade	0.521	**0.054**	**0.007**	**0.023**
	Planting × Shade	0.070	0.779	**0.051**	0.593
Production	Planting	0.968	0.468	0.257	**<0.001**
	Shade	**0.026**	0.711	**0.039**	0.073
	Planting × Shade	0.542	0.820	0.745	0.352
Leaf specific weight	Planting	0.102	0.637	**0.024**	0.262
	Shade	**0.051**	0.699	0.166	0.128
	Planting × Shade	0.624	0.515	0.097	0.070
Height	Planting	0.707	0.245	0.523	0.140
	Shade	**<0.001**	**<0.001**	0.092	0.440
	Planting × Shade	0.109	0.089	0.894	0.857

Table S2. P-values of two-way ANOVA for treatment effects and interaction on growth characteristics of *Heracleum moellendorffii* and *Adenophora divaricata* grown in low and high soil moisture. Significant p-values are written in bold font.

		Heracleum moellendorffii		*Adenophora divaricata*	
Parameter	Factor	Low moisture	High moisture	Low moisture	High moisture
Aboveground biomass	Planting	0.235	0.063	0.716	0.441
	Shade	0.545	0.503	0.969	0.620
	Planting × Shade	0.546	0.406	0.473	0.091
Belowground biomass	Planting	0.078	0.147	**0.027**	0.089
	Shade	0.556	0.581	0.307	0.548
	Planting × Shade	0.669	0.624	**<0.001**	0.639
Height	Planting	**0.018**	0.069	0.679	0.485
	Shade	0.129	**0.017**	0.075	0.598
	Planting × Shade	0.625	0.227	0.478	0.886
